# Post-COVID-19 condition: a sex-based analysis of clinical and laboratory trends

**DOI:** 10.3389/fmed.2024.1376030

**Published:** 2024-06-07

**Authors:** Carlos Delfino, M. Cecilia Poli, Cecilia Vial, Pablo A. Vial, Gonzalo Martínez, Amy Riviotta, Catalina Arbat, Nicole Mac-Guire, Josefina Hoppe, Cristóbal Carvajal, Paula Muñoz Venturelli

**Affiliations:** ^1^Centro de Estudios Clínicos, Instituto de Ciencias e Innovación en Medicina (ICIM), Facultad de Medicina Clínica Alemana Universidad del Desarrollo, Santiago, Chile; ^2^Facultad de Medicina Clínica Alemana de Santiago, Universidad del Desarrollo, Santiago, Chile; ^3^Programa de Inmunogenética e Inmunología Traslacional, Instituto de Ciencias e Innovación en Medicina, Facultad de Medicina, Santiago, Chile; ^4^Unidad de Inmunología y Reumatología, Hospital Roberto del Río, Santiago, Chile; ^5^Programa de Hantavirus y Zoonosis, Instituto de Ciencias e Innovación en Medicina, Facultad de Medicina, Santiago, Chile; ^6^Departamento de Enfermedades Cardiovasculares, Pontificia Universidad Católica de Chile, Santiago, Chile; ^7^Centro de Informática Biomédica, Instituto de Ciencias e Innovación en Medicina (ICIM), Facultad de Medicina Clínica Alemana—Universidad del Desarrollo, Santiago, Chile; ^8^Servicio de Neurología, Departamento de Neurología y Psiquiatría, Clínica Alemana de Santiago, Facultad de Medicina Clínica Alemana—Universidad del Desarrollo, Santiago, Chile; ^9^The George Institute for Global Health, Faculty of Medicine, University of New South Wales, Sydney, NSW, Australia

**Keywords:** post COVID-19 condition, long COVID, COVID-19, sex-disaggregated, neurologic long-COVID-19

## Abstract

**Background and aim:**

Post-COVID-19 condition (PCC) encompasses long-lasting symptoms in individuals with COVID-19 and is estimated to affect between 31–67% of patients, with women being more commonly affected. No definitive biomarkers have emerged in the acute stage that can help predict the onset of PCC, therefore we aimed at describing sex-disaggregated data of PCC patients from a local cohort and explore potential acute predictors of PCC and neurologic PCC.

**Methods:**

A local cohort of consecutive patients admitted with COVID-19 diagnosis between June 2020 and July 2021 were registered, and clinical and laboratory data were recorded. Only those <65 years, discharged alive and followed up at 6 and 12 months after admission were considered in these analyses. Multivariable logistic regression analysis was performed to explore variables associated with PCC (STATA v 18.0).

**Results:**

From 130 patients in the cohort, 104 were contacted: 30% were women, median age of 42 years. At 6 months, 71 (68%) reported PCC symptoms. Women exhibited a higher prevalence of any PCC symptom (87 vs. 60%, *p* = 0.007), lower ferritin (*p* = 0.001) and procalcitonin (*p* = 0.021) and higher TNF levels (*p* = 0.042) in the acute phase compared to men. Being women was independently associated to 7.60 (95% CI 1.27–45.18, *p* = 0.026) higher risk for PCC. Moreover, women had lower return to normal activities 6 and 12 months.

**Conclusion:**

Our findings highlight the lasting impact of COVID-19, particularly in young women, emphasising the need for tailored post-COVID care. The lower ferritin levels in women are an intriguing observation, warranting further research. The study argues for comprehensive strategies that address sex-specific challenges in recovery from COVID-19.

## Background

Since the declaration of the COVID-19 pandemic in March 2020, approximately 760 million individuals worldwide have been diagnosed with SARS-CoV-2 infection (1). Beyond the acute phase of the illness, some people experience ongoing symptoms, known as post-COVID-19 condition (PCC). PCC includes individuals with confirmed or probable COVID-19 who continue to have symptoms or develop new ones at least 3 months after the initial infection, lasting for at least 2 months (2). Studies suggest that a staggering 31 to 67% of patients infected with SARS-COV-2 endure these post-acute sequelae (3).

Among the published findings related to PCC, a stark disparity emerges, with women facing a significantly higher risk compared to men (63.2% vs. 36.8%) (4). It has also been proposed that the severity of the acute infection and BMI (5) may increase the risk of developing PCC, although this remains a topic of ongoing debate (6). Both systemic inflammation and neuroinflammation, as well as microvascular injury and thrombosis are critical to COVID-19 pathobiology (7, 8). Among these, the NLRP3 inflammasome plays a prominent role, triggering the release of highly inflammatory cytokines (e.g., IL-1β and IL-18) (9). Activation by SARS-CoV-2 of this complex results in the downstream production of interleukin-6 and C-reactive protein (CRP) (10). Additionally, the central nervous system can initiate an immune response through inflammasome activation (11). Moreover, a common genetic polymorphism (NLRP3 rs10754555 variant) has been reported to enhance systemic inflammation and inflammasome activity in patients with atherosclerosis, with those with the C/G and G/G genotype being at higher risk (12). This polymorphism may potentially influence the severity of COVID-19 and the neurological symptoms experienced by affected individuals. As of now, no biomarkers have emerged during acute COVID-19 that can predict the occurrence of PCC (13).

Because of the described sex predisposition to PCC, in this study, we sought to describe clinical and immunological profiles of acute COVID-19 patients, focusing on sex-specific analysis and potential predictors of PCC including comprehensive acute inflammatory and immunological response.

## Methods

### Study design, patients, and endpoints definitions

These analyses are based on a prospective single-centre cohort study conducted at Clínica Alemana Santiago, Chile. Patients under 65 years of age who were admitted for COVID-19 between June 2020 and July 2021 (corresponding to the two first waves of the pandemic) were consecutively enrolled. During this initial phase of the pandemic, where clinical assessments were severely restricted and there was a risk of underreporting comorbidities, we made the decision to concentrate on a younger demographic. This approach aimed to mitigate potential comorbidities that could independently contribute to poorer outcomes. During this period, the predominant circulating variants were Gamma (51.7%), Lambda (22.8%), and Alpha (6%) (14). Only patients who were discharged alive were included in the follow-up at 6 and 12 months. Detailed records of their previous medical history and acute clinical data upon admission were collected. Acute information regarding the patients was gathered during the initial 11 days of their hospitalization. The study protocol was approved by the local Ethics Committee (2022–33) and informed consent from all participants was obtained.

Baseline clinical-laboratory parameters including white blood cell count, ESR, CRP, ferritin and procalcitonin were measured at the time of acute hospital admission. In addition, acute phase samples were collected for comprehensive inflammatory response assessment including quantification of serum amyloid levels, inflammatory cytokines (IL-1β, IL-6, IL-8, IL-10, IL-12, IL-18, TNF) and chemokines (CCL2, CCL5, CCL8, CXCL9, CXCL10). Furthermore, samples were tested for the presence of the NLRP3 polymorphism (variant rs10754555), considering the C/G and G/G alleles as risk genotypes (12).

Following discharge, assessments were conducted by telephone interviews at 6 and 12 months to identify the presence of PCC symptoms using a structured questionnaire. These assessments utilized a structured questionnaire encompassing cognitive, cardiovascular, and gastrointestinal symptoms, as well as fatigue levels and return to normal activities. (Supplementary Table S1). Questions were related to current symptoms, therefore only those patients who still had symptoms at the time of the call were considered in the PCC group.

### Statistical analysis

Quantitative variables are reported as means ± SD or median (IQ range) depending on the normality (K-S test) and were compared using *T* Test or Mann–Whitney U test. Qualitative variables are reported as absolute and % prevalence and compared using the χ^2^ test or Fisher’s exact test. A multivariable logistic regression analysis was performed to explore variables associated with PCC. The variables were identified by univariate logistic regression analysis, including those that correlated significantly with the symptoms at follow-up and clinically significant variables were also included. In this analysis, we considered sex, age, BMI, data of acute care clinical setting, and comorbidities. Multivariable logistic regression was done to obtain an adjusted odds ratio with a 95% confidence interval. STATA version 18.0 was used to perform the analyses.

## Results

**Figure 1:**
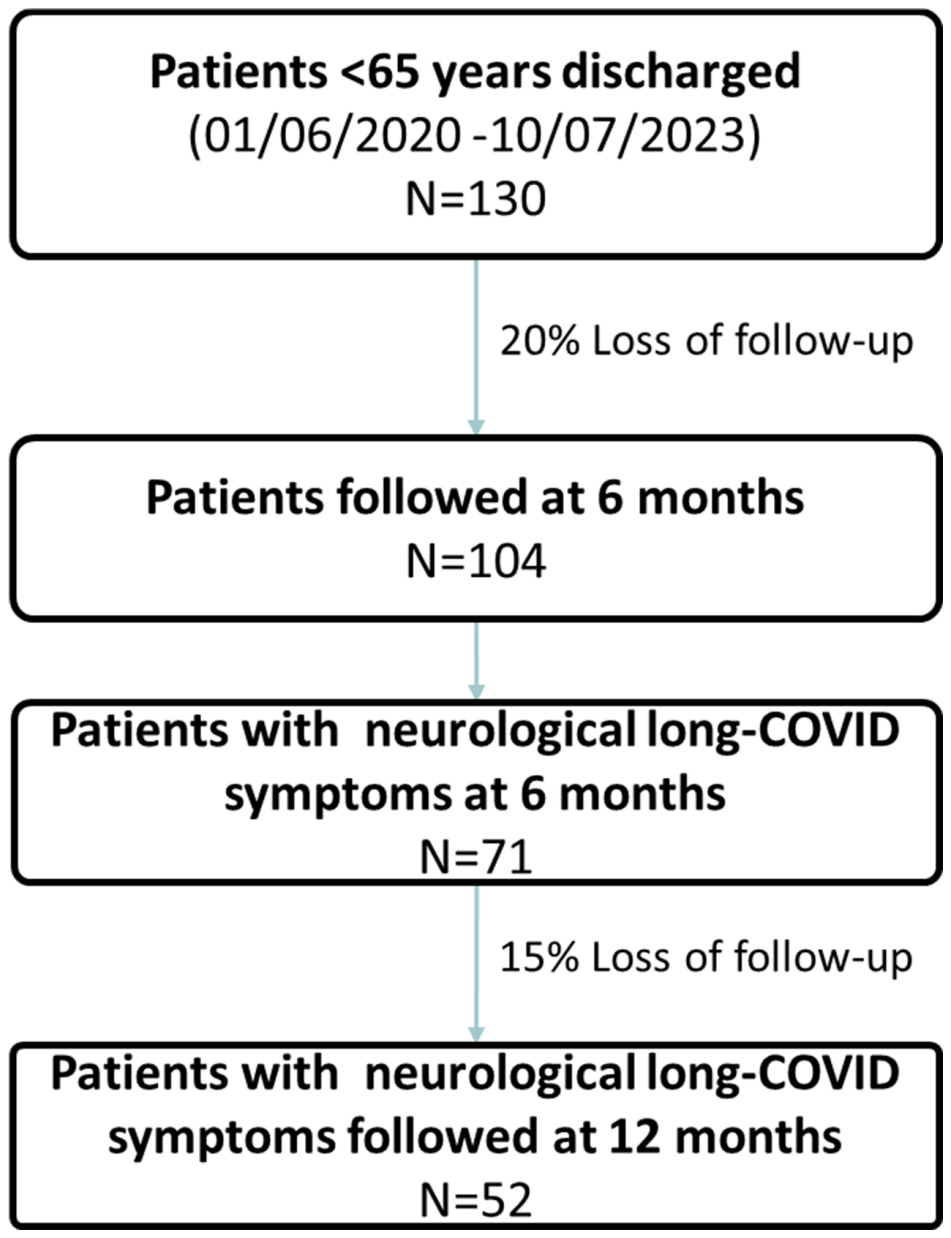
Study flowchart.

During the study period, a total of 130 patients under 65 years were discharged alive (Figure 1). At 6 months, 104 patients completed the follow up assessment. Patients had a median age of 42 years (IQR 37–56) and 30% were women (Table 1). Most of them had no comorbidities (64%), while a minority had been previously vaccinated against COVID-19 (19%), and only 11% required invasive mechanical ventilation (IMV). Regarding the acute laboratory findings and immune biomarkers obtained during acute hospitalization, it was noted that women had significantly lower ferritin values compared to men (465 vs. 1,141 ng/mL *p* = 0.004). No differences were found for inflammatory cytokines, chemokines or the presence of the NLRP risk variant (Table 1 and Supplementary Figures S1–S3).

**Table 1:** Demographic, clinical characteristics and inflammatory parameters of study participants.

	Total	Women	Men	*p*
*N* (%)	104	31 (30)	73 (70)	
Age, years*	42 (37–56)	44 (35–59)	41 (37–55)	0.741
BMI (kg/cm2)* (*n*=89)	27.99 (25.81–30.83)	27.63 (25.39–31.82)	28.27 (26.29–30.39)	0.921
Charlson Comorbidity Index=0 (no comorbidity), (*n*, %)	67 (64)	20 (64.5)	47 (64.3)	0.99
Length of hospitalization in days^*^	5 (4–6)	4 (3.5– 6.5)	5 (4–6)	0.855
IMV requirement (*n*, %)	11 (11)	3 (9)	8 (11)	0.846
Vaccination before the 6–month call (*n*, %)	20 (19)	6 (19)	14 (19)	0.983
Blood exams during hospitalization^*^				
WBC, /mm3	7450 (0.597–10200)	7100 (4500–1020)	7800 (6200–10200)	0.272
VHS (mm/h)	43 (27–58)	38 (24–56)	44 (29–60)	0.315
Highest value of CRP (mg/L)	2.64 (1.4–4.75)	3.08 (1.35–5.2)	2.63 (1.42–4.60)	0.762
Ferritin (ng/mL, *n*=77)	1010 (453–1722)	465 (236–1261.55)	1141 (700–1805)	0.004
Procalcitonin (ng/mL, *n*=79)	0.06 (0.02–0.09)	0.07 (0.04–0.11)	0.09 (0.06–0.16)	0.109
Serum Amiloide (mg/L)	327.32 (116.39–954.25)	265.8 (74.92–265.8)	446.6 (131.5–973.4)	0.584
Inflammatory cytokines during hospitalization^*^				
IL-1b (pg/mL)	4.82 (4.19–5.30)	4.52 (4.21–5.15)	4.86 (4.17–5.46)	0.288
IL-6 (pg/mL)	9.12 (6.26–19.89)	13.18 (6.64 –21.27)	8.23 (6.04–16.89)	0.198
IL-8 (pg/mL)	15.89 (11.49–25.76)	20.81 (12.43–26.36)	14.77 (11.35–24.30)	0.228
IL-10 (pg/mL)	4.29 (2.27–6.02)	4.07 (2.33–5.35)	4.36 (2.16–6.39)	0.596
IL-12 (pg/mL)	0.82 (0.40–1.47)	0.83 (0.34–1.17)	0.83 (0.42–1.49)	0.283
IL-18 (pM)	12.44 (9.18–16.62)	12.24 (7.21–15.94)	12.46 (9.45–18.06)	0.156
TNF (pg/mL)	0.12 (0–0.58)	0.26 (0–0.74)	0.09 (0–0.37)	0.136
Chemokines during hospitalization^*^				
CCL2 (pg/mL)	72.67 (42.89–119.68)	80.75 (51.57–122.49)	63.33 (37.77–119.59)	0.207
CCL5 (pg/mL)	17148.09	18016.27	16903.78	0.8
	(11475.71–25592.55)	(10798.15–25275.10)	(11592.01–25909.99)	
CXCL8 (pg/mL)	8.33 (5.30–19.27)	11.11(5.85–19.88)	7.91 (5.08–17.56)	0.346
CXCL9 (pg/mL)	173.18 (79.76–298.26)	170.34 (67.42–249.31)	192.18 (84.87–308.11)	0.399
CXCL10 (pg/mL)	544.20 (322.43–1118.59)	589.43 (363.46–1208.57)	519.84 (292.96–954.78)	0.567
Risk NLRP3 genotype^**^ (*n*, %)	64 (62)	18 (58)	46 (63)	0.774

^*^Values expressed as median and interquartile range (IQR), BMI: body mass index, IMV: Invasive mechanical ventilation; WBC, white blood cells; CRP, C reactive protein. ^**^C/G and G/G alleles were considered risk genotypes.

At 6 months, 71 out of 104 patients (68%) met the criteria for PCC, with a higher proportion of women (87 vs. 60%, *p* = 0.007) (Table 2). Of relevance, significant differences were observed between sexes. Women reported higher presence of cognitive (52 vs. 25% *p* = 0.007), cardiovascular (26 vs. 10% *p* = 0.031), and gastrointestinal (32 vs. 8% *p* = 0.022) symptoms compared to men. The evaluation of return to usual activities revealed a noteworthy gap: only 61% of women managed to resume their normal routines, whereas a substantial 90% of men with PCC achieved the same (*p* < 0.001). A comparison between those with and without PCC revealed a higher proportion of women in the former group (27 vs. 4, *p* = 0.007), as well as a greater requirement for IMV (11 vs. 0, *p* = 0.017) (Table 2). Nevertheless, no significant differences were observed in other clinical characteristics or blood test results (Table 2 and Supplementary Figures S4–S6). Moreover, 97% of individuals in the non-PCC group successfully resumed their usual activities compared to 75% within the PCC group (*p* = 0.006). In the group without PCC symptoms at 6 months, there were no significant differences between men (29/33) and women (4/33).

**Table 2:** Relevant acute phase characteristics and reported symptoms at 6 months.

a. Sex-based differences in symptom profiles at 6 months’ follow-up				
	Women (*n* = 31)	Men (*n* = 73)	*p*	Total (*n* = 104)
Any symptoms referred at 6 months (PCC), *n* (%)	27 (87)	44 (60)	0.007	71 (68)
Cognition	16 (52)	18 (25)	0.007	34 (32)
Fatigue	16 (52)	27 (37)	0.166	43 (41)
Cardiovascular	8 (26)	7 (10)	0.031	15(14)
Gastrointestinal	10 (32)	6 (8)	0.022	16 (15)
Return to daily duties at 6 months	19 (61)	66 (90)	< 0.001	66 (90)

b. Differential analysis of demographic and laboratory characteristics at 6 months: patients with and without PCC						
	With PCC (*n* = 71)				Without PCC (*n* = 33)	*p* ^***^
	Women (*n* = 27)	Men (*n* = 44)	*p* ^**^	Total		
Age, years^*^	45 (36–59)	43 (37.5–55)	0.669	44 (37–56)	40 (37–56)	0.216
BMI (kg/cm2) (*n* = 89)	27.06 (25.42–31.93)	28.40 (26.54–30.86)	0.744	28.19 (25.71–31.56)	27.98 (26.15–29.38)	0.364
Charlson Comorbidity Index = 0 (no comorbidity), (*n*, %)	17 (63)	28 (63)	0.955	45 (63)	22 (67)	0.745
Length of hospitalization in days	5 (4–7)	5 (4–7)	0.891	5 (4–7)	4 (4–6)	0.081
IMV requirement (*n*, %)	3 (11)	8 (18)	0.424	11 (15)	0	0.017
Vaccination before the 6-month call (*n*, %)	4 (14)	8 (18)	0.713	12 (17)	5 (15)	0.313
Blood exams during hospitalization^*^						
Ferritin (ng/mL)	470 (332–1190.8)	1,695 (849.75–2279.55)	0.001	1,062 (519–1805)	876.55 (340.351433)	0.178
Procalcitonin (ng/mL)	0.06 (0.05–0.12)	0.11 (0.08–0.18)	0.021	0.1 (0.06–0.16)	0 0.08 (0.04–0.12)	0.082
Seric Amiloide (mg/L)	265.8 (74.92–990.6)	379.17 (123.14–987.65)	0.549	311.74 (114.8–988.6)	363.36 (117.99–890.67)	0.880
TNF (pg/mL)	0.26 (0–1.38)	0 (0–0.312)	0.042	0 (0.10–0.59)	0.020 (0–0.62)	0.203

PCC: Post COVID Condition; BMI: body mass index; IMV: Invasive mechanical ventilation. ^*^values expressed as median and interquartile range (IQR). ^**^ *p*-values reflect comparisons between men and women with PCC. ^***^ *p*-values comparing total patients with and without PCC.

**Figure 2:**
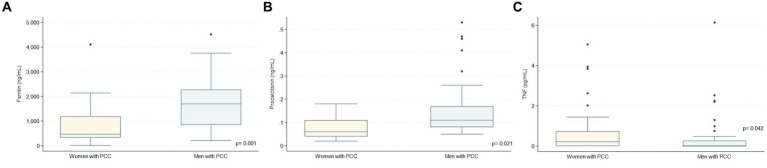
Acute phase biomarkers during hospitalisation according to sex in patients with PCC symtoms at 6  months. * PCC: post COVID-19 condition. **(A)** Ferritin, **(B)** Procalcitonin, **(C)** TNF.

In the PCC group, there were differences regarding laboratory findings during hospitalisation between sexes: women exhibited lower levels of ferritin (470 vs. 1,695 ng/mL, *p* = 0.001) and procalcitonin (0.06 vs. 0.11, *p* = 0.021), but higher TNF values (0.26 vs. 0, *p* = 0.042) compared to men in the acute phase (Table 2; Figure 2; Supplementary Figures S4–S6). Being women was the only independent predictor factor for PCC at 6 months, as they were 7.60 times more likely to experience it compared to men (*p* = 0.026, CI 1.27–45.18, Supplementary Figure S7).

At 12 months, 87% of patients with previous PCC at 6 months still had symptoms but showed no evident clinical or laboratory differences by sex (Supplementary Table S2). Importantly, in this subgroup, only 56% of women were able to return to their regular activities, as opposed to 86% of men (*p* = 0.004).

## Discussion

The results of our study provide valuable information on the lasting impact of COVID-19 among adults under the age of 65 with non-critical disease. Despite a higher initial admission rate of men for COVID-19, PCC affected predominantly women. Specifically, they reported a higher prevalence of cognitive, cardiovascular, and gastrointestinal compromise. This is in line with previous reports (15–17), although it is noteworthy that these women did not have other concurrent comorbidities, as has been observed in other cohorts (18).

Only female sex was found to be predictive of subsequent PCC. This finding is consistent with previous research, which highlights the notable association between the risk of PCC and specific socio-demographic factors, in particular female sex (19). Although some studies have hinted at possible links with ethnicity or pre-existing conditions (such as poor mental and general health or asthma), there is a lack of consistent evidence across studies to designate these as reliable predictors of PCC (20–22). Despite this, we observed clear acute differences in ferritin and procalcitonin levels between sexes, with lower levels in women than in men. Many studies have found a link between elevated ferritin levels and increased risk of death. However, the relationship is complex, and other factors can play a role (19, 20). It should be noted that, to our knowledge, no previous research has specifically examined sex disparities in ferritin values among patients with mild COVID-19. However, the lower ferritin values observed in women could be attributed to the fact that they experience a milder acute infectious course. In addition, women showed higher TNF values than men. This is consistent with recent studies that have indicated elevated TNF levels in patients with post-COVID symptoms, suggesting its potential role as a predictor of PCC (21). This finding could be related to variations in immune response, hormonal factors, or other underlying biological mechanisms. The absence of notable disparities in inflammatory cytokines, chemokines and the NLRP3 risk variant suggests a more nuanced interaction between sex and immune response in COVID-19. At 12-month follow-up, we observed that patients with PCC had no significant clinical or laboratory differences, suggesting a possible stabilisation or stagnation of symptoms in this subgroup, possibly influenced by different factors such as the initiation of COVID-19 vaccination (20).

In terms of the return to daily activities, when comparing individuals with and without PCC, the PCC-affected group demonstrated greater difficulty resuming their usual routines (75% vs. 97%, *p* = 0.006). Within the PCC group, women showed significantly lower rates of resumption of usual activities compared to men, both at 6-and 12-months follow-up. This observation points to a possible impact on quality of life and highlights the specific obstacles that women may encounter during their recovery process. This may be associated with a higher prevalence of neuropsychiatric symptoms (23) and the societal expectation that males often shoulder the primary role in household support. It underlines the need for personalised care plans after COVID-19, especially adapted to female patients.

The results herein support the need to establish PCC assessment in all adults in the aftermath of COVID-19, particularly in women, as predictive factors in the acute setting remain elusive.

To the best of our knowledge, this represents the largest cohort of COVID-19 patients with a 12-month follow-up, coupled with a comprehensive evaluation of inflammatory biomarkers. This is especially significant as obtaining blood samples during the early stages of the pandemic posed considerable challenges, given the limited availability of specific laboratory reagents and the associated costs of analysis. Notably, this cohort primarily comprised individuals affected during the two initials waves of the pandemic; therefore, effects of infection can be assessed independently of vaccination, which could be confounding.

Our study has remarked limitations that deserve to be acknowledged. First, it is a single-centre investigation conducted in a relatively uniform cohort of patients with moderate COVID-19 severity, because of challenges associated to consenting acute severe patients for the study or had died at follow up. In addition, participants were under 65 years old. Therefore, larger scale studies covering a broader spectrum of patients, including those who did not require hospitalisation and with more comorbidities, are essential to validate these findings. Second, our admission information was limited to 11 days, potentially leading to loss of relevant information from the acute phase. However, the comprehensive characterisation of acute patients, including assessment of inflammatory markers and evaluation of risk genotypes, lends strength to the study results. Finally, discharge follow-up was conducted by telephone and employing a concise questionnaire with broad questions regarding PCC symptoms, which could introduce bias in the results by restricting participation to those who could answer the call and incomplete information. Throughout the pandemic, numerous studies have employed similar methodologies, demonstrating their reliability (22–24). Unlike other studies with high non-response rates or unreachable participants, our study had only a 20% dropout rate at 6 months and a 15% dropout rate at 12 months (25). Nevertheless, it is likely that our results are more representative of a younger, healthier population, whereas frail subjects are under-represented in our study.

## Conclusion

In summary, our study emphasizes the significance of acknowledging and addressing sex-specific nuances among COVID-19 survivors. These findings support the need for a more individualized and comprehensive approach to post-COVID care, with particular attention to the distinct challenges encountered by female patients. Further research is essential to elucidate the underlying mechanisms contributing to these disparities and to enhance interventions for achieving the best possible recovery and rehabilitation outcomes.

## Data availability statement

The datasets presented in this study can be found in online repositories. The names of the repository/repositories and accession number(s) can be found at: https://www.ncbi.nlm.nih.gov/clinvar/, SUB14168930.

## Ethics statement

The studies involving humans were approved by Centro de bioética, Facultad de Medicina Clínica Alemana, Universidad del Desarrollo. The studies were conducted in accordance with the local legislation and institutional requirements. The participants provided their written informed consent to participate in this study.

## Author contributions

CD: Data curation, Formal analysis, Validation, Writing – original draft, Investigation. MCP: Investigation, Supervision, Writing – review & editing. CV: Investigation, Methodology, Writing – review & editing. PVi: Conceptualization, Supervision, Writing – review & editing. GM: Conceptualization, Supervision, Visualization, Writing – review & editing. AR: Data curation, Investigation, Project administration, Writing – review & editing. CA: Writing – review & editing, Investigation. NM-G: Writing – review & editing, Investigation. JH: Writing – review & editing, Investigation. CC: Software, Writing – review & editing. PMV: Formal analysis, Funding acquisition, Investigation, Methodology, Project administration, Resources, Writing – original draft, Writing – review & editing.
