# Supplementary material for: Post-COVID-19 condition: a sex-based analysis of clinical and laboratory trends
Source: Front Med (Lausanne). 2024 Jun 7;11:1376030. doi: 10.3389/fmed.2024.1376030 (PMC11198115; doi:10.3389/fmed.2024.1376030)
Supplement: Supplementary file 1 [file Data_Sheet_1.docx]

Supplementary Material

**Post-COVID-19 Condition: A Sex-Based Analysis of Clinical and Laboratory Trends**

Carlos Delfino, Cecilia Poli, Cecilia Vial, Pablo Vial, Gonzalo Martínez, Amy Riviotta, Catalina Arbat, Nicole Mac-Guire, Josefina Hoppe, Cristóbal Carvajal, Paula Muñoz Venturelli^.^

- Supplementary tables:2
- Supplementary figures: 7

**Supplementary tables**

**Table S1**. Structured questionnaire applied at 6 months.

|  |  | Yes | No | Comment |
| --- | --- | --- | --- | --- |
| **Cognitive** | |  |  |  |
|  | Compared to your pre-COVID-19 state, do you feel that you have worse concentration or forget things easier? |  |  |  |
| **Fatigue** | |  |  |  |
|  | Do you get more tired during physical activity than before COVID-19? |  |  |  |
| **Cardiovascular** | |  |  |  |
|  | Have you experienced new or more tachycardia or palpitations when you are at rest compared to your pre-COVID state?? |  |  |  |
| **Gastrointestinal** | |  |  |  |
|  | Have you developed new food intolerances and/or recurrent diarrhoea and/or constipation compared to your pre-COVID state? |  |  |  |
| **Return to usual activities** | |  |  |  |
|  | Have you been able to return to the usual activities you did before COVID-19? |  |  |  |

| **Supplementary Table 2.** Post COVID-19 Condition (PCC) symptoms reported at 12 months | | | | |
| --- | --- | --- | --- | --- |
|  | **Total** | **Women** | **Men** | **p** |
| **N (%)** | 52/60 (87) | 23/24 (96) | 29/36 (56) | 0.088 |
| **Symptoms refered at 12 months, n (%)** | | | | |
| Cognition | 30 (58) | 16 (70) | 14 (48) | 0.123 |
| Fatigue | 33 (63) | 14 (61) | 19 (65) | 0.730 |
| Cardiovascular | 12 (23) | 7 (30) | 5 (17) | 0.262 |
| Gastrointestinal | 9 (17) | 6 (26) | 3 (10) | 0.136 |
| **Return to daily duties** | 35 (67) | 12 (52) | 23 (79) | **0.038** |
| PCC: Post COVID-19 Condition | | | |  |

**Supplementary Figures**

**Supplementary Figure 1.** Blood tests during hospitalisation of study participants (n=104)


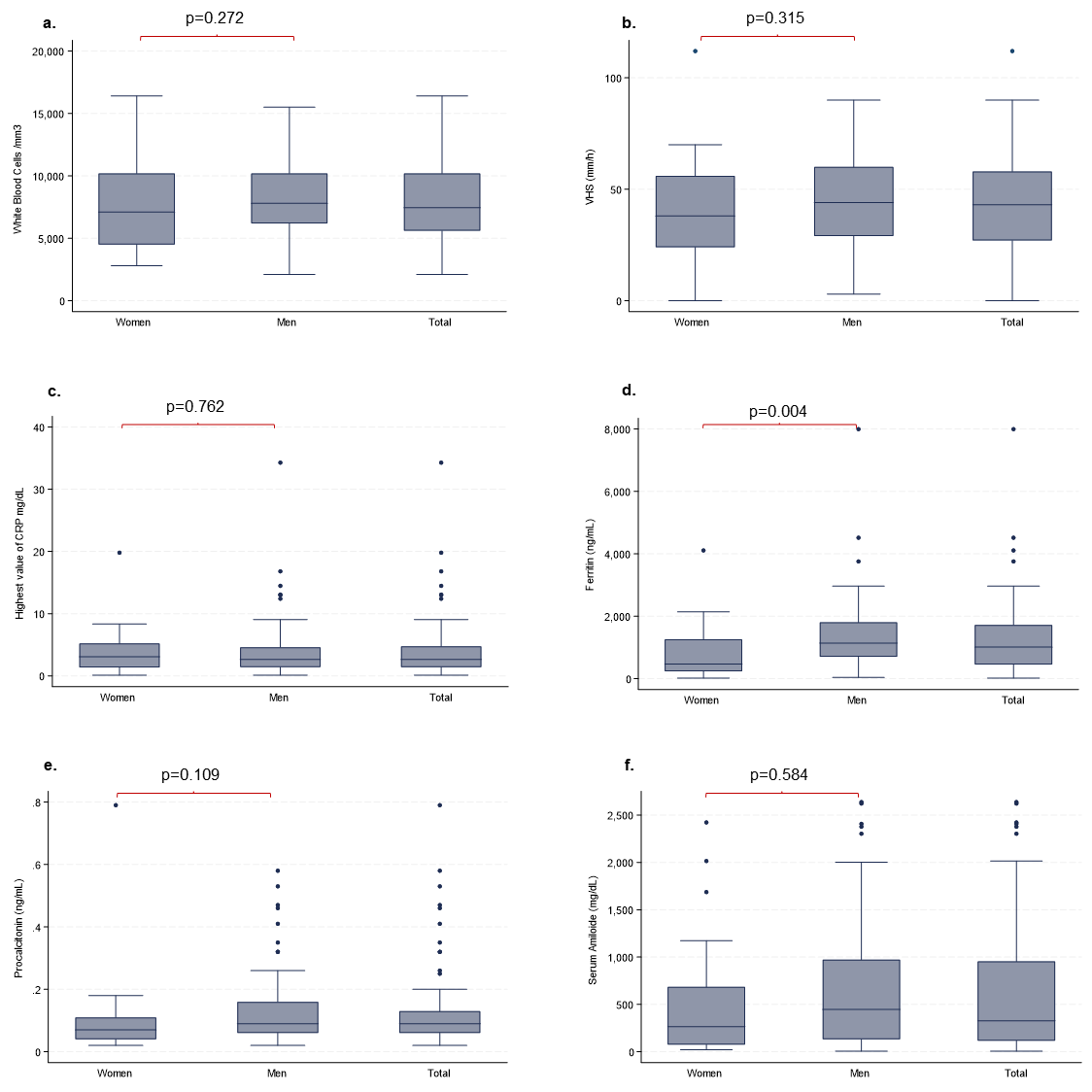


**Supplementary Figure 2**. Inflammatory cytokines during hospitalisation of study participants (n=104)


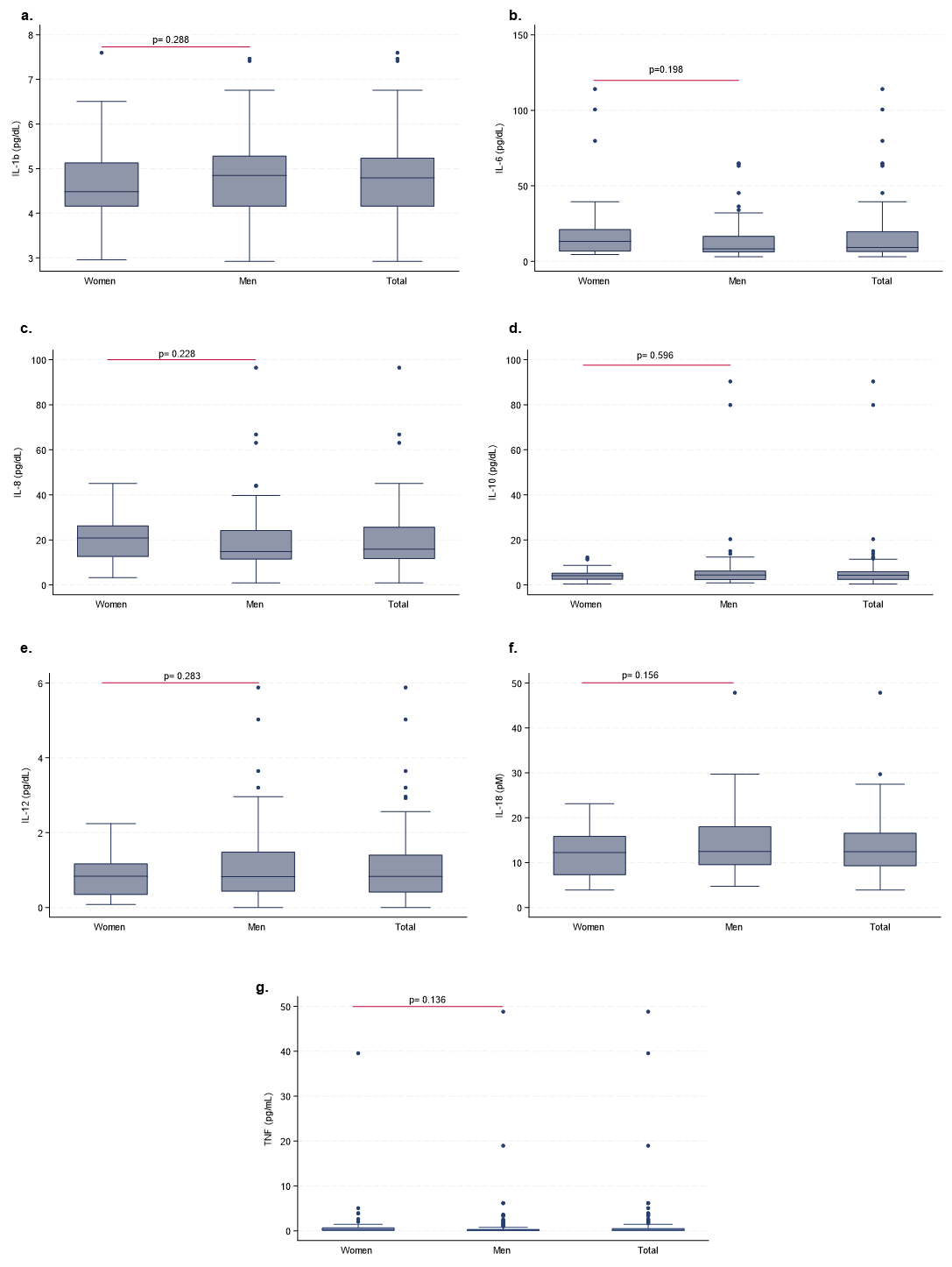


**Supplementary Figure 3.** Chemokines during hospitalisation of study participants (n=104)

**b.**

**a.**

**c.**

**d.**

**e.**

**Supplementary Figure 4.** Comparison of blood test results during hospitalisation: Participants with and without Post-COVID-19 condition, including sex breakdown in the affected group.

**b.i**

**b.ii**

**a.i**

**a.ii**

**c.i**

**c.ii**

**d.i**

**d.ii**

**e.i**

**e.ii**

*Post-COVID-19 condition

**Supplementary Figure 5**. Comparison of inflammatory cytokines during hospitalisation: Participants with and without Post-COVID condition, including sex breakdown in the affected group.
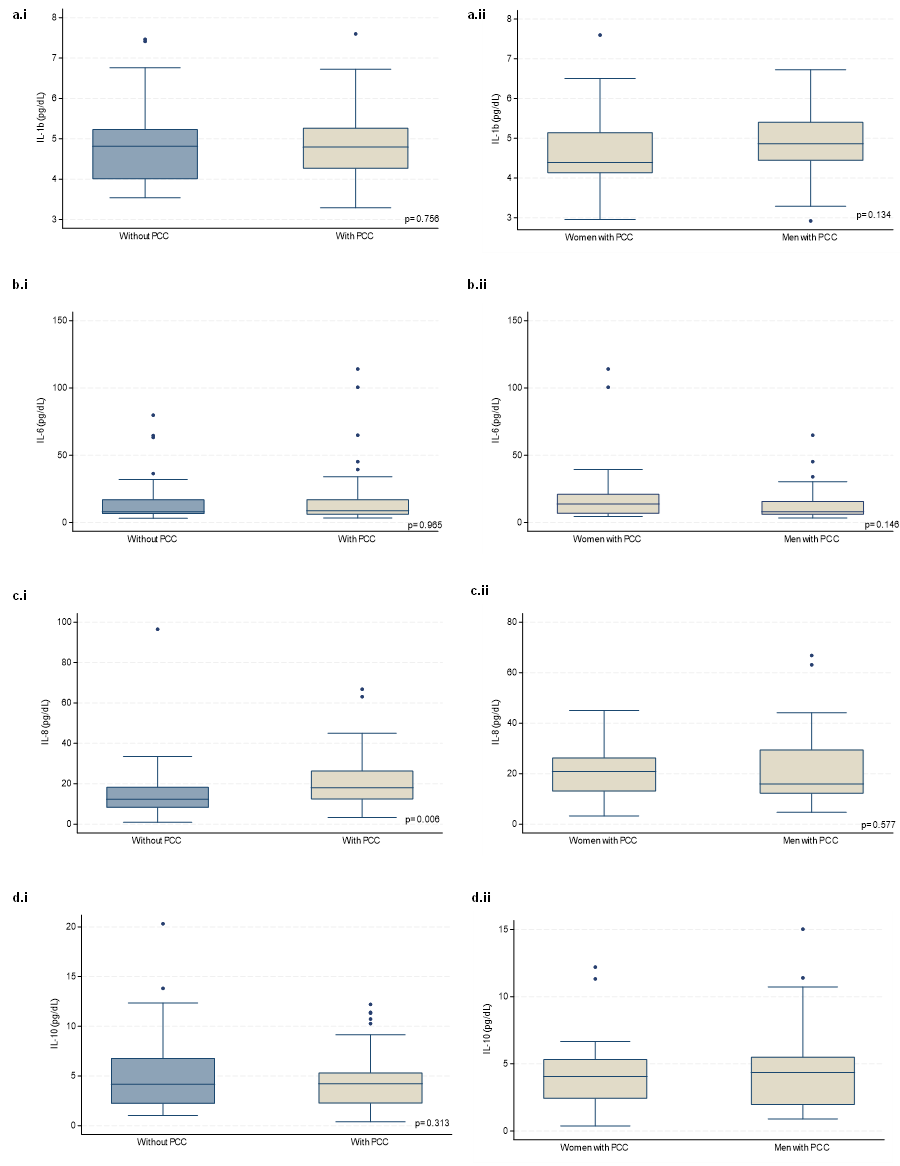


(Cont Fig 5)


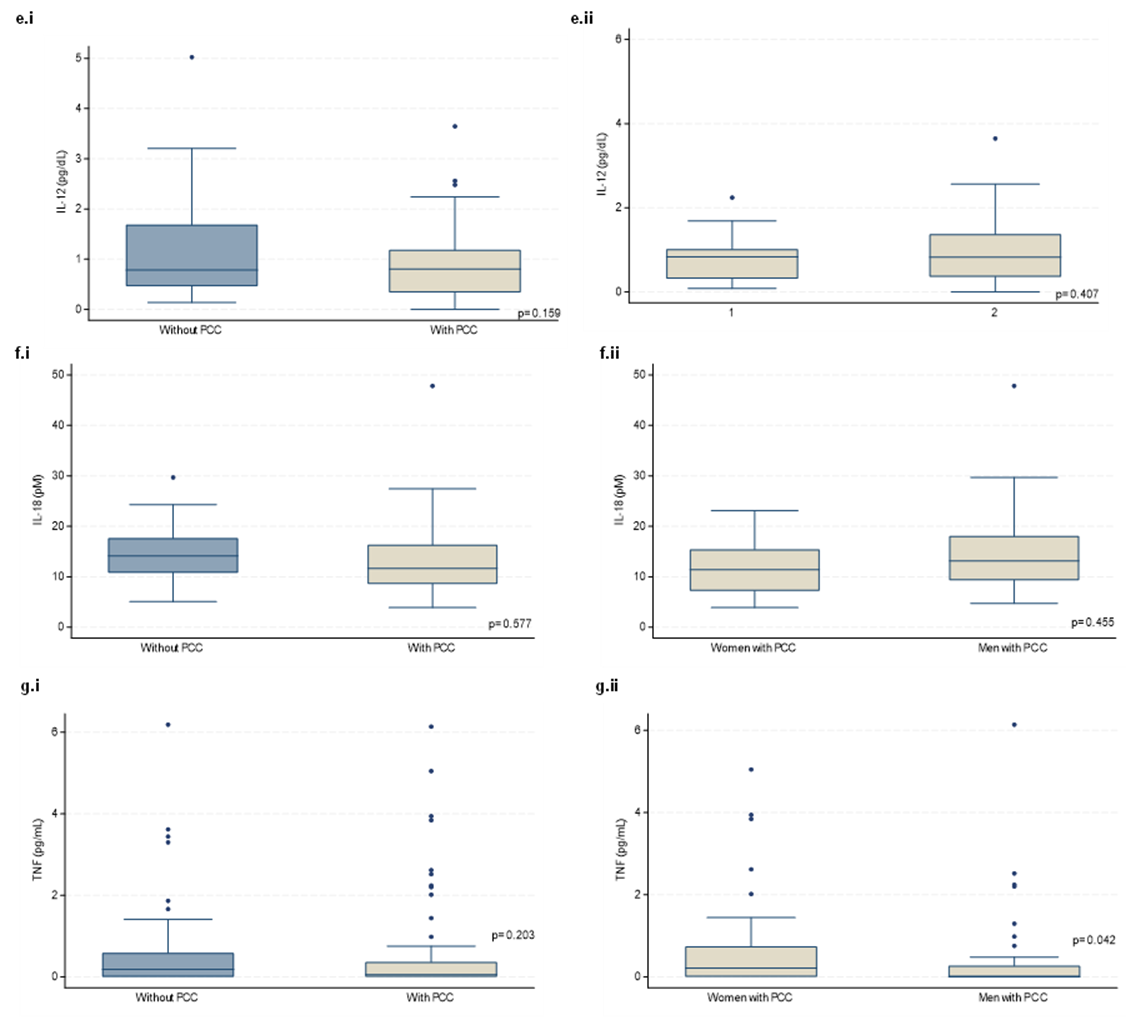


*Post-COVID-19 condition

**Supplementary Figure 6.** Comparison of inflammatory chemokines during hospitalisation: Participants with and without Post-COVID-19 condition, including sex breakdown in the affected group.


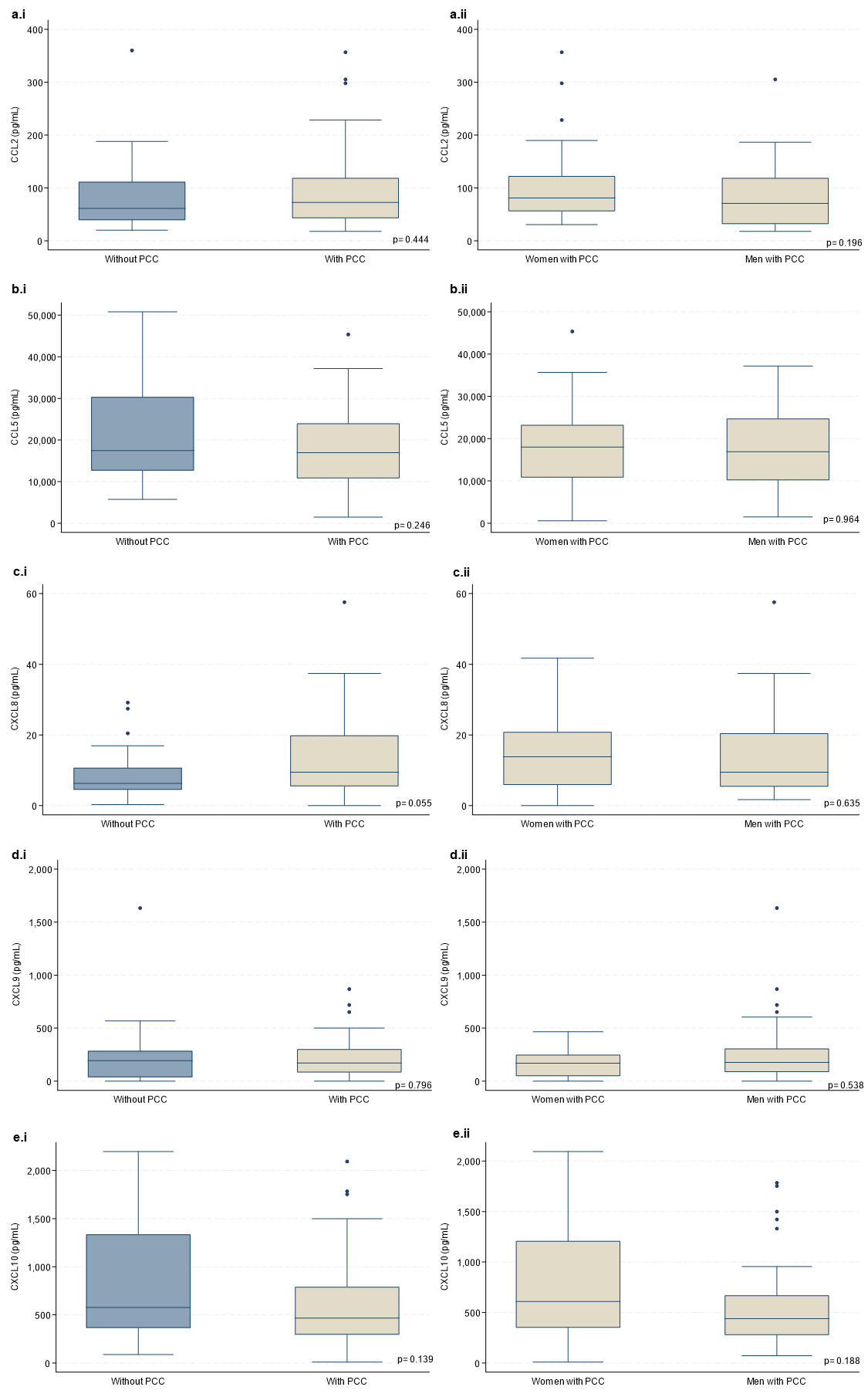


*Post-COVID-19 condition


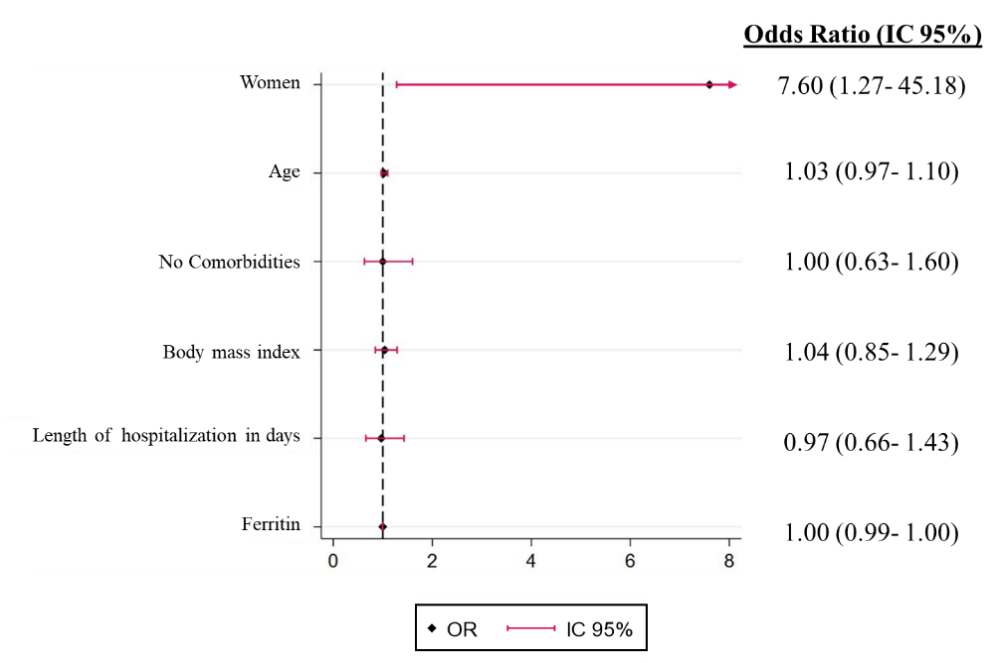
**Supplementary Figure 7.** Predictors of Post COVID-19 condition (PCC) at 6 months
